# Increasing PRIDE Skills for Child Engagement Using CDI-Only iPCIT with Children with Autism Spectrum Disorder

**DOI:** 10.3390/bs16050662

**Published:** 2026-04-28

**Authors:** Jordan T. Etherington, Zachary C. LaBrot, Emily R. DeFouw, Brad Dufrene, Brittany D. Garza, Amarah Sweaks, Briley Newcomb

**Affiliations:** 1 School of Psychology, College of Education and Human Sciences, The University of Southern Mississippi, Hattiesburg, MS 39406, USA; zachary.labrot@usm.edu (Z.C.L.); emily.defouw@usm.edu (E.R.D.); brad.dufrene@usm.edu (B.D.); brittany.garza@usm.edu (B.D.G.); amarah.sweaks@usm.edu (A.S.); brileynewcomb@usf.edu (B.N.); 2 Department of Educational and Psychological Studies, College of Education, The University of South Florida, Tampa, FL 33620, USA

**Keywords:** autism, parent training, iPCIT, child engagement, challenging behavior, social validity

## Abstract

Many children with autism spectrum disorder (ASD) display elevated levels of challenging behaviors and decreased social engagement. Parent Child Interaction Therapy (PCIT) may help children with ASD increase rates at which they engage and interact with their caregivers. Existing barriers to treatment completion, including high attrition and travel time and costs, may be ameliorated by delivering PCIT virtually (iPCIT) and setting attainable mastery criteria. This study utilized a multiple baseline design across three caregiver-child dyads with ASD to determine whether an iPCIT protocol affected caregivers’ use of positive engagement skills, children’s engagement, and children’s challenging behaviors. Results demonstrated increased and improved caregiver-implemented engagement and behavior management practices with some improvements in child outcomes. These findings suggested that iPCIT is an effective treatment modality for teaching parents to increase and maintain their use of positive engagement skills across time. Without explicit instructions to avoid negative interaction behaviors, increases in positive engagement skills did not result in concomitant decreases in negative interaction behaviors. Increases in social engagement were observed for two children, but a decrease in engagement was observed in the third. Implications for future research and practice are discussed.

## 1. Introduction

Autism spectrum disorder (ASD) is characterized by deficits in social communication and interaction and restricted or repetitive patterns of behavior, interests, or activities (American Psychiatric Association, 2022). For example, many children with ASD show delays in joint attention, imitation, gesturing, or using facial expressions. Further, although not a diagnostic feature, many children with ASD display challenging behaviors, such as aggression, tantrums, self-injurious behaviors, and defiance (Eisenhower et al., 2005; Mahan & Matson, 2011). Interventions targeting ASD focus on improving communication and interaction skills and decreasing challenging behaviors. Behavioral therapies are considered a frontline approach for both challenging behaviors and ASD and target key processes that maintain challenging behaviors (Comer et al., 2013).

The coercive parenting process, also called the coercive family process (Lunkenheimer et al., 2016; Patterson, 2002), proposes that challenging behaviors are maintained when a child has been reinforced for engaging in such behavior in the past. For example, when a parent tries to address these behaviors, their child may respond with increased intensity or frequency of disruptive behavior. In response, the parent may withdraw the pressure on the child to try to stop the behaviors, which can inadvertently reinforce the child’s intensified challenging behavior. If the behaviors continue to escalate, they may continue to inadvertently reinforce cyclically escalating behavior (Patterson, 2002). Behavioral parent training seeks to alter the antecedents and consequences of behavior to reverse this cycle.

### 1.1. Parent–Child Interaction Therapy

Based on behavioral principles, social learning theory, and attachment theory, Parent–Child Interaction Therapy (PCIT) is one intervention that targets the coercive parenting process to reduce challenging behaviors (Williford et al., 2018). PCIT consists of a child-led, play-based phase (child-directed interaction [CDI]) where parents learn skills to follow their child’s lead during play, followed by a parent-led phase (parent-directed interaction; [PDI]) where parents are taught to set boundaries on behavior and implement appropriate and consistent discipline for boundary violations (Shawler & Funderburk, 2018). A unique and critical component of PCIT is the provision of live coaching to parents during both phases, which allows for immediate feedback, shaping of parent skills, and direct observation of learning progress (McNeil & Hembree-Kigin, 2010; McNeil et al., 2019). Although PCIT has an extensive research base supporting its effectiveness for treating challenging behaviors (Cooley et al., 2014; Thomas et al., 2017; Ward et al., 2016), less literature has examined the effects of PCIT on other socially and clinically relevant behavioral outcomes (e.g., child engagement).

Given its interactive nature, it is possible that PCIT may also improve deficits in social engagement. Specifically, PCIT teaches caregivers to use five positive interaction skills called PRIDE skills (i.e., praise, language reflections, imitations, descriptions of behavior, and enjoyment/enthusiasm in the interaction). Each of the PRIDE skills provides caregiver attention to the child’s behaviors, which may reinforce social behaviors (McNeil & Hembree-Kigin, 2010). For example, providing praise for or imitating desired play activities may increase the frequency in which the child engages in those or similar behaviors. Attention can also be provided to shape emerging social behaviors by successively providing attention for closer approximations of social behaviors, such as communicating with the caregiver or sharing toys. In addition, praise statements, language reflections, and behavioral descriptions provide children with models of positive social interactions that they can imitate.

Previous work examining social engagement in the context of PCIT has been limited to the narrower construct of joint engagement as an outcome of PCIT (Furukawa et al., 2018; Han, 2021; Rigg, 2016). Joint engagement refers to interactions in which a parent and child are actively involved with a common event or object at the same time and plays a role in the development of language, literacy, perspective-taking, and false-belief understanding (Adamson et al., 2014; Nelson et al., 2008). Further, Bottema-Beutel (2016) found that variations in joint attention abilities, a precursor to joint engagement in which the child shares looks, points, or gestures with another person (Adamson & Dimitrova, 2014), were more closely linked with receptive and expressive language in children with ASD compared to typically developing children. These studies (Furukawa et al., 2018; Han, 2021; Rigg, 2016) demonstrated mixed results, with limited experimental control. Specifically, Han (2021) reported medium-to-large effect sizes for improvements in joint engagement, but the study was underpowered due to a limited sample size, and treatment integrity was not reported. Rigg (2016) reported an increase in joint attending from a child with various adults after intervention was implemented in one setting but no further changes when the intervention was introduced in additional settings. Finally, although parents in Furukawa et al. (2018) reported anecdotal improvements in their children’s joint engagement, the study did not systematically measure joint engagement, and parents were instructed to implement an additional intervention targeting joint attention, confounding the effects of PCIT alone. To address the limitations of the existing research, studies need to be experimental in nature, able to demonstrate treatment fidelity and experimental control, measure social engagement behaviors more broadly, and have sufficient power to detect differences if using a group design.

### 1.2. PCIT for ASD

PCIT has specifically been shown to decrease disruptive child behaviors and parent stress, and to increase parent’s use of PRIDE skills, in families with children with ASD (Vetter, 2018). Further, a recent analysis of PCIT in families with children with ASD showed that children demonstrated similar reductions in challenging behaviors regardless of ASD severity, use of psychiatric medication, receptive language ability, or age (Allen et al., 2022).

Given the interactive nature of PCIT and the social deficits frequently demonstrated in children with ASD, PCIT may also ameliorate some of the core symptoms of ASD. Several studies to date have demonstrated improvements in ASD-related symptoms following PCIT, including atypicality, hyperactivity, aggression, withdrawal, inattention, pretend play, verbalizations, physical touch, communication, social responsiveness, social awareness, restricted/repetitive behaviors, prosocial skills, adaptive skills, and expressive language (Lieneman et al., 2018; Parladé et al., 2020; Scudder et al., 2019; Vess & Campbell, 2022; Zlomke & Jeter, 2020; Zlomke et al., 2017). In order to realize these benefits, families must be able to appropriately and effectively receive treatment.

Previous work has demonstrated high levels of attrition in PCIT. Estimates suggest that only 32–73% of families complete PCIT (Jent et al., 2021). Ufford et al. (2022) reported that high mastery criteria, long treatment lengths, travel distances, and high costs were among the reasons cited by parents who terminated PCIT services prematurely. As such, ameliorating these barriers may improve PCIT attrition. Lieneman et al. (2019) showed that many families who attended as few as four CDI sessions reported significant improvements in child challenging behaviors, suggesting that mastery criteria could be decreased and the length of PCIT shortened while still providing treatment benefits to families. Because treatment effects may be apparent as early as the fourth CDI session and because opportunities for appropriate social interaction are primarily taught within the CDI phase (e.g., PRIDE skills as reinforcers and models), it is possible that the CDI phase alone may be beneficial for children with autism. Providing the CDI phase of PCIT without the PDI phase may decrease levels of challenging behavior (Lieneman et al., 2019) and increase social engagement while directly addressing concerns related to high mastery criteria and long treatment lengths.

Further, as caregivers increase their use of PRIDE skills, caregivers might naturally use fewer “Don’t skills,” particularly because PRIDE skills and “Don’t skills” are incompatible responses (McNeil et al., 2019). If instruction related to “Don’t skills” is omitted from PCIT, caregivers may be able to increase their focus on PRIDE skills to promote positive interactions. Further, providing PCIT virtually using live, web-based communication software (iPCIT) may decrease travel and cost barriers for families who live far away from treatment facilities.

### 1.3. iPCIT

iPCIT offers several advantages over in-person PCIT. Videoconferencing with families in their homes reduces or eliminates travel barriers, reduces child reactivity—allowing the clinician to observe and respond to more realistic behaviors, simplifies skill transfer to the home setting, and improves attendance (Barnett et al., 2021). Families receiving iPCIT, compared to traditional PCIT, have reported fewer treatment barriers generally (Comer et al., 2017).

Because PCIT is traditionally facilitated by the clinician in a separate room from the parent and child, few changes need to be made to the standard PCIT protocol to deliver iPCIT (Comer et al., 2017). For example, instead of providing coaching through a one-way mirror and “bug-in-the-ear,” the clinician provides coaching through a webcam and Bluetooth earpiece. iPCIT has been successfully implemented in several empirical demonstrations (Comer et al., 2017; Fleming et al., 2021; Pogue, 2022), with comparable outcomes as traditional PCIT.

Research on iPCIT for children with ASD is limited to two case studies (Hong et al., 2018; Kawasaki & Kamo, 2020) and one experimental study (Matano et al., 2022). Hong et al. (2018) reported that a 3-year-old who lived too far from an in-person intervention facility demonstrated improvements in challenging behaviors and communication following iPCIT. Kawasaki and Kamo (2020) reported an increase in parent PRIDE skills and a decrease in child challenging behaviors following iPCIT for a child suspected of having ASD and attention-deficit hyperactivity disorder (ADHD). Matano et al. (2022) reported similar levels of improvement for the one family who received iPCIT compared to three families who received traditional PCIT in person. However, each of these studies contained methodological and practical limitations. Specifically, maintenance or follow up data after the conclusion of iPCIT was only collected by Hong et al. (2018); studies lacked or had limited experimental control, studies collected only indirect reports of child behaviors with no objective data on child engagement, data on parents’ use of skills were missing from some studies, and it is unclear from these studies whether an increase in PRIDE skills can result in a decrease in “Don’t skills” without direct training. Further research is needed to address these limitations in the extant literature.

### 1.4. Present Study

The aim of this study was to determine whether iPCIT is an effective treatment to increase child engagement for children with ASD by experimentally implementing iPCIT using a multiple baseline design across participants. While the research cited above has demonstrated the efficacy of PCIT for this population, attrition rates for PCIT are high due to barriers such as costs and distance of travel. If effective, iPCIT may ameliorate these barriers to treatment and allow families greater access to treatment. To this end, the following eight research questions were developed:

1. Is there a functional relation between CDI-only iPCIT and an increase in caregiver PRIDE skills?
2. Do increases in PRIDE skills maintain after iPCIT sessions end?
3. Is there a functional relation between CDI-only iPCIT and a decrease in caregiver “Don’t skills” if caregivers are not taught to avoid them?
4. Is there a functional relation between iPCIT and an increase in child engagement in children with ASD?
5. Do higher rates of child engagement maintain after iPCIT sessions end?
6. Does CDI-only iPCIT result in a decrease in caregiver-reported challenging behaviors in children with ASD as measured by the ECBI?
7. Do caregivers perceive PRIDE skills as a socially valid strategy to improve child joint engagement?
8. Do caregivers perceive iPCIT as a socially valid parent training modality?

## 2. Materials and Methods

### 2.1. Participants and Setting

Three caregiver-child dyads were recruited from treatment waitlists at community and university behavioral health centers in the Southeastern United States. Families were eligible to participate if they had a child between the ages of 2 and 7 years old with a clinical diagnosis of ASD (verified through a review of records) and the caregiver was able to speak and understand English. Families were excluded from the study if they had previously received or were currently receiving behavioral parent training or if the child displayed severe aggression that warranted more intensive intervention. Pseudonyms are used to refer to the participants throughout the paper.

The first dyad consisted of Jacob (child) and Hannah (caregiver). Jacob was a 2 ½-year-old Caucasian male who was diagnosed with ASD. Jacob communicated primarily using gestures (e.g., pointing, handing objects to Hannah) and some single-word utterances and received speech therapy services throughout the duration of the study. Hannah was Jacob’s biological mother, a Caucasian female who identified herself as Deaf and used external hearing aids to understand spoken language. Hannah communicated with Jacob using both English and American Sign Language. Hannah was a single parent who shared a household with her parents and sister. Hannah had a bachelor’s degree at the time of the study.

The second dyad was Anthony (child) and Candance (caregiver). Anthony was a 4-year-old African American male who was diagnosed with ASD and ADHD. Anthony communicated primarily using gestures (e.g., pointing, moving objects or Candance’s hands towards or away from stimuli) and some single-word utterances. Anthony also received speech therapy services throughout the course of the study. Additionally, between the final session of the intervention phase and the first session of the maintenance phases of the present study, Anthony began receiving occupational therapy services and began taking guanfacine prescribed by his pediatrician for ADHD; the dose and regularity at which Anthony took the medication were unknown. Candance was Anthony’s biological mother, an African American female who was married with 6 children, of which Anthony was the fourth. Candance had a bachelor’s degree and stayed home to care for her children.

The third dyad consisted of Luke (child) and Melissa (caregiver). Luke was a 6-year-old Caucasian male diagnosed with ASD. Luke communicated in phrase speech (e.g., “draw tablet”) and short sentences (e.g., “I’m all done with big boy school”) to communicate his wants and needs and engage in social conversation with Melissa. Although Luke received speech therapy at school, Luke and Melissa’s participation in the study occurred during the summer months, when Luke was not actively receiving speech therapy services. Luke was not receiving any other therapy services during the study. Melissa was Luke’s noncustodial paternal grandmother, a Caucasian female. Luke lived with his biological parents and paternal grandparents. Both of Luke’s parents had college or advanced degrees and are employed at jobs with inconsistent hours, so Melissa, who was retired, often provided care for Luke during the day. During the intervention phase, after the “CDI Teach” session but before any data collection sessions had occurred, Melissa experienced a fall that resulted in fractures in both of her arms, which limited her mobility throughout the remainder of the study. In addition, Luke’s mother attended the first four observation sessions during the intervention phase so that she could assist Melissa if Luke engaged in any aggressive behavior; however, Luke did not exhibit any aggressive behavior in any of the sessions his mother attended.

The interventionist and primary researcher was a White, male doctoral student in his 3rd year of graduate training in school psychology, originally from a Western US State. The graduate student received introductory training in PCIT delivery via an online training module (UC Davis CAARE Center, 2023; Sacramento, CA, USA) and was supervised by a licensed psychologist with expertise in the behavioral treatment of children with ASD and other neurodevelopmental disorders.

All data collection and training sessions occurred via Zoom (version 6.1; San Jose, CA, USA), a HIPAA- and FERPA-compliant videoconferencing software program (FERPA Guide, 2020; HIPAA Compliance Datasheet, 2021). The interventionist logged into the videoconferencing software from a private office, while the participants logged into the videoconferencing software from living or playrooms in their homes.

### 2.2. Instruments and Materials

#### 2.2.1. Behavior Intervention Rating Scale

The Behavior Intervention Rating Scale (BIRS; Elliott & Treuting, 1991) was given to each caregiver at the conclusion of the intervention phase to assess their perception of PRIDE skills as a behavior management technique. The BIRS consists of 24 Likert-type items ranging from 1 (strongly disagree) to 6 (strongly agree). Previous research on the BIRS has demonstrated adequate reliability and validity (Elliott & Treuting, 1991), with a three-factor structure: acceptability (63% of variance), effectiveness (6% of variance), and time of effectiveness (4.3% of variance). The α coefficient for the BIRS was 0.97, which suggests high internal consistency. α coefficients for the acceptability, effectiveness, and time of effectiveness subscales were 0.97, 0.92, and 0.87, respectively. For this study, the word “teacher” was changed to “parent.” Descriptive statistics (e.g., mean, range, standard deviation) for each factor of the BIRS were analyzed for each participant.

#### 2.2.2. Clinical Consultation Acceptability and Satisfaction Scale

The Clinical Consultation Acceptability and Satisfaction Scale (C-CASS; LaBrot & Kupzyk, 2018) was given to each caregiver at the conclusion of the intervention phase to assess their perceptions of the acceptability, appropriateness, and effectiveness of the treatment procedures. The C-CASS comprises 12 Likert-type items; scores range from 0 (strongly disagree) to 5 (strongly agree), with higher scores indicating greater treatment acceptability. The C-CASS is adapted from the Consultation Acceptability and Satisfaction Scale (CASS; Dufrene & Ware, 2018) to make it applicable to a clinical setting. For example, the word “teachers” was changed to “parents,” and the words “classroom practices” were changed to “behavior management practices” (LaBrot & Kupzyk, 2018). The CASS has shown high internal consistency, with an α coefficient of 0.978 (Dufrene & Ware, 2018). Descriptive statistics (e.g., mean, range, standard deviation) from the C-CASS were analyzed for each participant.

#### 2.2.3. Eyberg Child Behavior Inventory

The Eyberg Child Behavior Inventory™ (ECBI; Eyberg & Pincus, 1999) is a 36-item caregiver-report measure of challenging behaviors. The ECBI was administered to caregivers digitally by sending a link from the publisher’s website to the caregivers’ email addresses. The ECBI was administered at the beginning and end of the study. For each item (e.g., argues about rules, temper tantrums, lies), the caregiver reported how frequently their child engages in the behavior on a 7-point Likert-type scale from 1 (never) to 7 (always) and whether it is a problem for them. Scores from each item were summed to the Intensity and Problem scales, respectively. Scores are considered clinically significant if they are above 132 on the Intensity scale and 15 on the Problem scale. Psychometric analysis for the ECBI in children with neurodevelopmental disorders has revealed good reliability and validity of the measure in this population when used in both research and practice settings (Jeter et al., 2017; Martinez et al., 2022). Descriptive statistics from the ECBI were analyzed for each factor (i.e., problem and intensity) across time for each participant, where available.

### 2.3. Dependent Measures and Data Collection Procedures

#### 2.3.1. PRIDE Skills

The primary dependent variable for this study was caregivers’ use of PRIDE skills. PRIDE skills refer to the positive parent–child interaction skills which were taught to caregivers in the “CDI Teach” session and coached throughout the remainder of the intervention. They included behavior-specific praise, reflective statements, imitations, and behavioral descriptions (Dufrene et al., 2024). Definitions for these behaviors were modified from the original DPICS-IV definitions (Eyberg et al., 2014) to make them more appropriate for children with ASD.

Labeled praise was operationally defined as any specific verbalization that expressed a positive evaluation of an activity, product, or attribute; labeled praise must have been response dependent, and must have specifically referenced a specific activity, product, attribute, and/or behavior (Dufrene et al., 2024). Examples of labeled praise included, “You’re so good at brushing the baby’s hair,” and, “You built a tall tower, great job!” Non-examples of labeled praise included, “You’re so pretty,” and, “You built a taller tower than me.”

A reflection referred to a phrase or statement that repeated the child’s immediately preceding verbalization or vocalization; reflections may have been the exact same words/vocalization the child said, contained synonymous words, or contained some elaboration upon the child’s statement, but the basic content must have been the same as the child’s message or sound similar to the child’s verbalization (Dufrene et al., 2024). Examples of reflective statements included a caregiver who said, “You want me to do it again,” after a child approximated the word “again,” or a caregiver who said, “Yes, that is a beautiful horse,” after a child says, “pretty pony.” A non-example of a reflective statement included, “I like both,” after the child asked, “Should I make cereal or pancakes?” During the “CDI Teach” session, the clinician and caregiver discussed specific vocalizations the child used that were appropriate to reflect, based on the child’s language abilities. For example, since Jacob spoke using only a few single-word utterances, Hannah was instructed to reflect any one-word utterances, word approximations, or other meaningful vocalizations Jacob used.

Imitation was operationally defined as any physical action by the caregiver that physically copied or mimicked a child’s physical action (within 5 s); the imitation may have been the exact same physical action the child made, contained some similar actions, or contained some additional actions, but the basic physical action must have been similar to something the child did (Dufrene et al., 2024). For example, a child was stacking blocks, and the caregiver started stacking blocks 2 s later, even if different blocks were used, or the stacks were shaped differently. A non-example was a child stacking blocks, and the caregiver began rolling a toy car.

A behavioral description was operationally defined as a non-evaluative phrase or sentence in which the subject was the child, and the verb described the child’s ongoing or immediately completed (within 5 s) observable verbal or nonverbal behavior (Dufrene et al., 2024). An example of a behavioral description was, “You are eating a pancake” (while the child is eating a real or imaginary pancake). Nonexamples included, “You are thinking about TV” (non-observable behavior) and “you smiled when we got here today” (occurred more than 5 s ago).

Observations were conducted by the primary researcher, who was previously trained to code caregiver and child behaviors to a 90% agreement criterion. Data were collected from 5 min audiovisual recordings of caregiver-child play interactions, which were collected twice weekly. Caregivers’ rate of PRIDE skills was recorded using an event recording procedure where the frequency of each PRIDE skill was recorded within 10 s intervals and reported as the number of combined PRIDE skills used per 5 min observation.

#### 2.3.2. Don’t Skills

“Don’t skills” referred to the caregiver-led interaction skills which caregivers were taught to avoid. In this study, however, caregivers were not taught or coached to avoid the use of these skills. The “Don’t skills” included asking questions, giving instructions, and giving criticisms. A question was a verbal inquiry that began with an interrogative word or phrase and/or ended with rising inflection that requests an answer but did not suggest that a behavior was to be performed by the child (Eyberg et al., 2014). An example of a question was, “What do you want to play?” An instruction could have been direct or indirect and was an order, demand, or direction for a behavioral response from the child; indirect instructions may have been interpreted as optional, implied, or stated in question form, where a direct instruction was declarative and sufficiently specific to indicate which behavior was expected from the child (Eyberg et al., 2014). An example of an indirect instruction was, “Can you put the red block there?” and an example of a direct instruction was, “Look at mommy.” A nonexample of an instruction was, “What is your name?” (requires only a verbal response). Finally, a criticism was any statement directed toward a child that involved asking the child to stop a behavior (e.g., “Stop talking,” “Don’t do that”), corrective statements (e.g., “You shouldn’t do that”), or any verbal statement that called attention to disruptive behavior (LaBrot et al., 2021). An example of a criticism was, “Stop throwing toys,” while a nonexample was “I think that block is actually red.” Caregiver’s use of “Don’t skills” was measured using the same procedure as PRIDE skills. As with PRIDE skills, the frequency of each “Don’t skill” was recorded in 10 s intervals and reported as the number of combined “Don’t skills” used per 5 min observation.

#### 2.3.3. Child Engagement

Child engagement was defined as any attempt the child made to get, maintain, or direct their caregiver’s attention to objects or actions of their own interest. Examples of child engagement included giving objects to or taking objects from their caregiver, showing objects to their caregiver by placing objects within the caregiver’s eyesight, making vocalizations toward their caregiver, directing facial expressions (e.g., smiles, puzzlement) toward their caregiver, and engaging in back-and-forth, turn-taking interactions with the caregiver, including conversations. Nonexamples included undirected vocalizations (e.g., vocal stimming) or movements (e.g., motor stereotypy) and parallel play (i.e., child playing appropriately or interacting with toys independently). Child engagement was recorded using a momentary time sampling procedure (Saudargas & Zanolli, 1990) in which the observer checked whether the child was engaged at the end of each 10 s interval. Child engagement was reported as a percentage of intervals in each observation in which the child was engaged.

#### 2.3.4. Challenging Behavior

On the day of the first baseline observation, each caregiver was administered the ECBI to measure their perception of their child’s challenging behavior. Each caregiver was administered the ECBI a second time immediately following the final maintenance phase observation to measure changes in caregiver-reported child challenging behavior. Candance did not complete the second ECBI, so post-intervention challenging behavior data were not available for Anthony. ECBI scores were analyzed via descriptive statistics for quantitative differences before and after intervention for the participants who completed the ECBI at both time points.

#### 2.3.5. Social Validity

Immediately following the final data collection observation of the intervention phase, each caregiver was administered the BIRS and C-CASS. Both measures were administered via a single Qualtrics survey provided to the caregiver via email. Instructions provided within the Qualtrics survey informed caregivers that the BIRS asked questions related to the value of PRIDE skills as a behavior management technique, while the C-CASS asked questions relating to the virtual training process.

### 2.4. Experimental Design, Data Analysis, and Phase Change Decisions

A nonconcurrent multiple baseline design across participants was used to evaluate the effects of iPCIT on caregiver and child outcomes. The multiple baseline design is the most widely used experimental design in applied behavioral research, and experimental control is demonstrated in a multiple baseline design when a change in behavior is observed only at the time the experimental variable is applied to each participant (Cooper et al., 2020). This study included the following phases: (A) baseline, (B) intervention, and (C) maintenance. This study used a nonconcurrent design (Morin et al., 2024) because data collection began as participants were referred for intervention, which occurred at different points in time.

Observation data collected from this study were analyzed visually in regard to their level, trend, variability, immediacy of effect, data overlap across phases, and consistency of data patterns across phases (Horner et al., 2005). Descriptive statistics were used to quantify visual analysis. In addition to visual analysis, the Baseline Corrected Tau (BC-Tau) effect size (Tarlow, 2017) was calculated using the Single-case effect size calculator (version 0.7.3; Pustejovsky et al., 2024) to determine the amount of overlap between the baseline and successive phases. BC-Tau was selected due to its ability to correct for trends in the baseline phase (Tarlow, 2017). BC-Tau was interpreted via nonparametric effect size analysis according to Vannest and Ninci (2015).

Phase changes were determined by prespecified criteria for each transition, based primarily on caregiver PRIDE skills. Specifically, the intervention phase began for the first participant after at least six data points have been collected for each variable (i.e., PRIDE skills, “Don’t skills,” and child engagement) and when levels of PRIDE skills were relatively stable across observations or trending downward. Successive participants began intervention with a two-data-point stagger if they met the same criteria and the previous participant had demonstrated an intervention effect. The maintenance phase began after a minimum of six data collection sessions, when caregivers’ PRIDE skills demonstrated a clear intervention effect and showed stable or increasing levels of PRIDE skills, provided they had demonstrated a minimum of three of each PRIDE skill across three consecutive observations. Notably this differs from the traditional CDI mastery criteria of ten instances of each skill (Eyberg & Funderburk, 2022), in response to Ufford et al. (2022), who reported that high mastery criteria and the resulting length of the CDI phase are associated with high attrition. Additionally, exceptions were made for (1) Hannah’s reflections due to Jacob typically vocalizing fewer than three times per session (Scudder et al., 2019) and (2) Melissa’s imitations due to her limited mobility.

### 2.5. Procedures

#### 2.5.1. Intake

Prior to participating in the study, the primary researcher conducted a brief in-person intake session with caregiver-child dyads interested in participating in the study. The primary researcher verified that the dyad met inclusion criteria, briefly explained study procedures, and obtained informed consent.

#### 2.5.2. Baseline

During baseline, caregivers logged onto Zoom with the primary researcher for observations. Each observation lasted 5 min, and caregivers were instructed to interact with their child as they normally would. To decrease reactivity from the caregiver or child, the researcher turned off his video camera and microphone during all data collection sessions. Data were collected twice each week, with one to three successive recordings collected per session. Caregivers did not receive any instruction or feedback on PRIDE skills, “Don’t skills,” or child engagement during baseline.

#### 2.5.3. iPCIT

Immediately following the final baseline session, the “CDI Teach” session was conducted. During this caregiver-only session, the interventionist employed behavioral skills training (BST), a teaching package composed of instructions, modeling, rehearsal, and feedback that has been used effectively for individuals with neurodevelopmental disorders (Leaf et al., 2015). Specifically, the clinician instructed the caregiver on the use of PRIDE skills using both verbal and written instructions; caregivers were encouraged to refer to the written instructions as needed throughout play and data collection sessions. Video models (Lieneman, 2023) were presented to caregivers for each skill.

After caregivers had been provided instruction and modeling for all PRIDE skills, caregivers were shown another video of a child and caregiver playing to provide the caregiver opportunities to rehearse each of the PRIDE skills; the interventionist paused the video frequently and asked the caregiver to use a PRIDE skill as if they were playing with this child. If the caregiver used a PRIDE skill correctly, the interventionist provided the caregiver with behavior-specific praise and resumed the video. If the caregiver did not use a PRIDE skill correctly, the interventionist reviewed the components for the corresponding PRIDE skill with the caregiver and provided the caregiver with a correct response and prompted the caregiver to repeat the correct response or provide another example of a correct response. When the caregiver provided a corrected response, the interventionist resumed the video. The process was completed until each caregiver had practiced each PRIDE skill correctly a minimum of 5 times. Before the “CDI Teach” session ended, the interventionist and caregiver discussed the child’s language abilities and what kinds of vocalizations were appropriate to reflect based on the child’s language abilities. The “CDI Teach” session lasted between 45 and 60 min.

During successive CDI sessions, caregivers were instructed to play with their child while implementing the PRIDE skills. The interventionist provided positive and constructive feedback via live coaching. Coaching sessions lasted 20–30 min.

Modifications were made to the standard PCIT protocol (Eyberg & Funderburk, 2022) related to the experimental and telehealth nature of this study. Specifically, only the CDI phase of PCIT was implemented in the current study (Lieneman et al., 2019). Second, parents were not instructed to avoid the use of “Don’t skills” at any time during the study. Third, the mastery criteria were decreased from 10 labeled praise statements, 10 reflections, and 10 behavior descriptions to 3 labeled praise statements, 3 reflections, 3 imitations, and 3 behavior descriptions to determine whether decreasing the intensity of CDI still resulted in treatment effects (Ufford et al., 2022). In total, the intervention phase lasted three to four weeks.

Further modifications for the telehealth nature of the study were based on recommendations from Peskin et al. (n.d.) related to telehealth provision of PCIT. Specifically, sessions were conducted via videoconference, video models were used in place of live models, PCIT handouts were provided to caregivers digitally, the interventionist’s video camera was turned off during coaching to minimize reactivity from the child, and caregivers wore Bluetooth earpieces during coaching to allow them to hear the interventionist without impeding their ability to hear their child.

#### 2.5.4. Maintenance

The maintenance phase began on the first data collection day following the final CDI coaching session. The maintenance phase was conducted in the same manner as the baseline phase. Maintenance data were collected for a minimum of five observations.

### 2.6. Interobserver Agreement and Treatment Integrity

Secondary observers, consisting of one White, male, undergraduate student in psychology and two White, female, graduate students in school psychology, all of whom were trained to code caregiver and child data with at least 90% reliability, coded a minimum of 30% of sessions per phase (baseline, intervention, and maintenance) for each participant. Interobserver agreement (IOA) was calculated using an exact count-by-interval IOA (Cooper et al., 2020) for each variable. Thus, an interval was scored as an agreement only if both observers recorded the same number of each skill for each variable (i.e., the same number of labeled praise statements, reflections, imitations, and behavioral descriptions) in an interval. When IOA for any variable fell lower than 80% on a recording, both observers met, reviewed operational definitions and the recording, and came to a consensus on the codes for that video recording. Following this conferencing process, IOA for PRIDE skills averaged 95% (range: 80–100%), IOA for “Don’t skills” averaged 93% (range: 80–100%), and IOA for child engagement averaged 92% (range: 80–100%) for child engagement.

Treatment integrity was measured every session based on integrity checklists described in Eyberg and Funderburk (2022). Modifications to standard procedures are described above. Treatment integrity for the “CDI Teach” session averaged 99% (range: 97–100%) Treatment integrity for the coaching sessions averaged 98% (range: 91–100%) In addition, caregivers practiced PRIDE skills 68% (range: 14–100%) of days between sessions; the lowest rate of practice was between Melissa and Luke after Melissa first fractured her arms.

## 3. Results

### 3.1. Caregiver Skills

**Figure 1:**
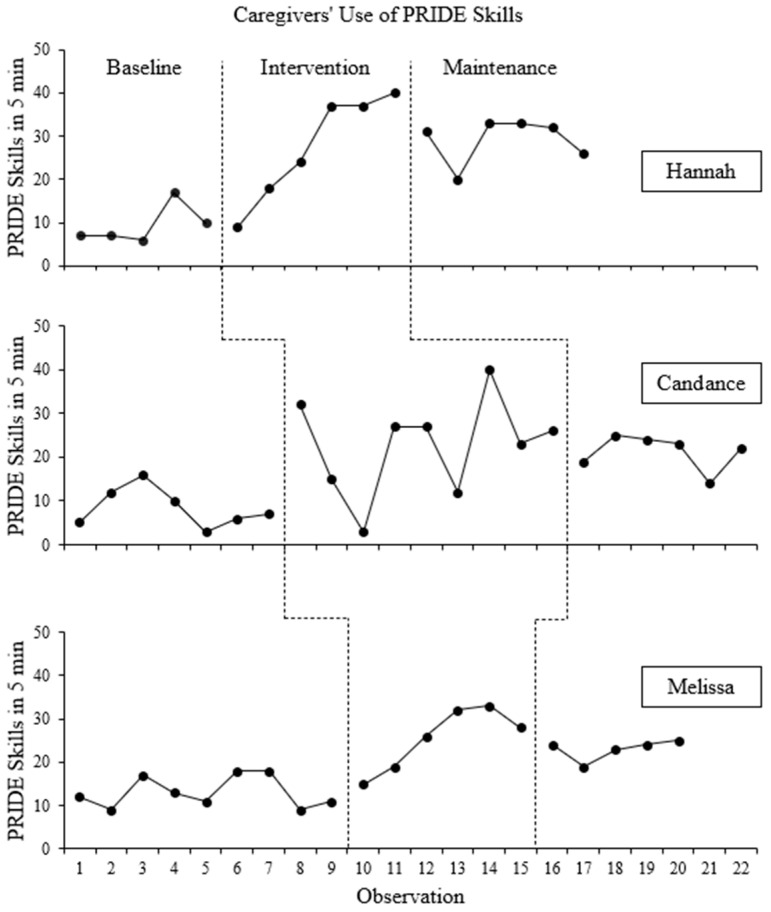
Caregivers’ Use of PRIDE Skills.

**Figure 2:**
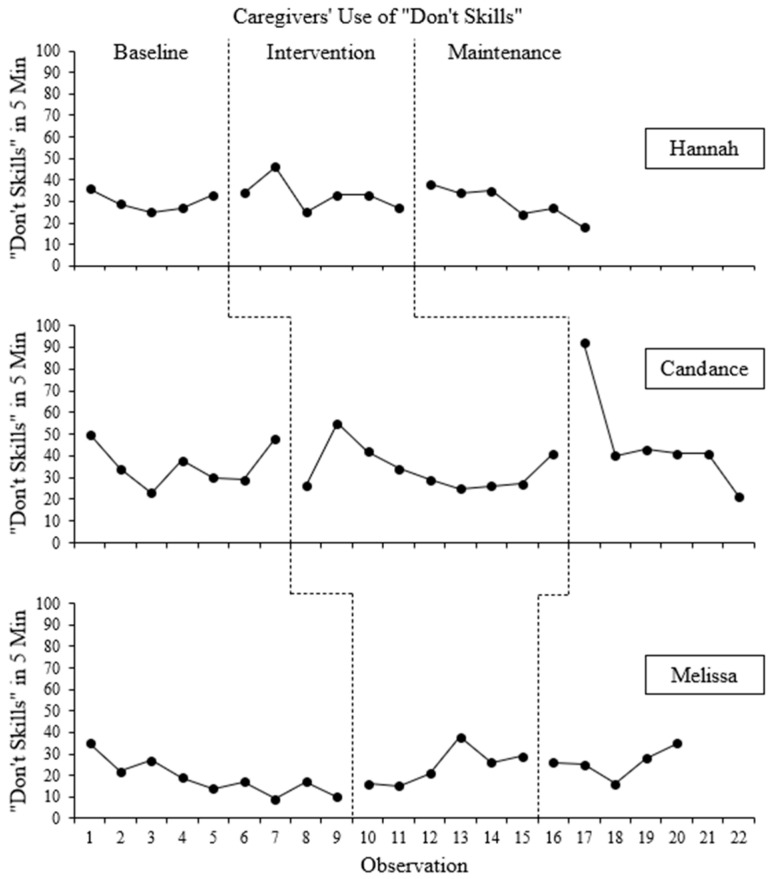
Caregivers’ Use of Don’t Skills.

**Figure 3:**
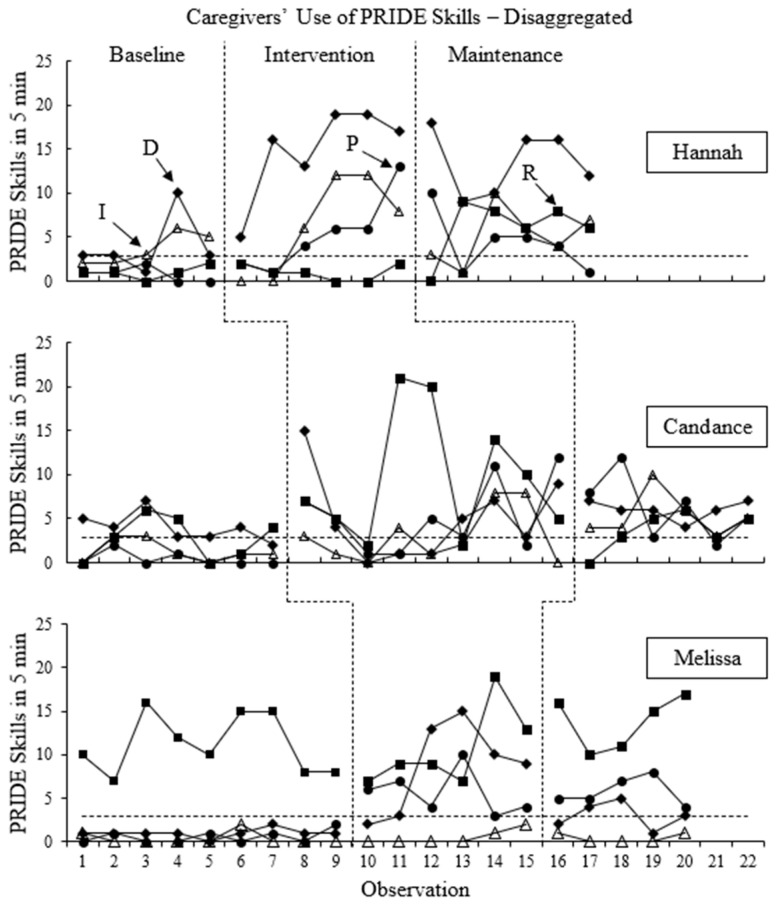
Caregivers’ Use of PRIDE Skills—Disaggregated. Note. P = Labeled Praise; R = Reflection; I = Imitation; D = Description. The horizontal dotted line represents three uses of each skill.

The frequency counts of PRIDE skills and “Don’t skills” used in each 5 min observation are presented for each participant in Figure 1 and Figure 2, respectively. Frequency counts for PRIDE skills are disaggregated in Figure 3.

#### 3.1.1. Hannah

Hannah used an average of 9.4 (range: 6–17) PRIDE skills per 5 min session during baseline. Hannah demonstrated some variability in the number of PRIDE skills during baseline, with a slight overall increasing trend across the baseline phase. During the intervention phase, there was a steady increase in Hannah’s use of PRIDE skills, which averaged 27.5 (range: 9–40) PRIDE skills. Although there was not an immediate increase in level, Hannah’s use of PRIDE skills increased throughout the intervention phase, and only the first data point overlapped with baseline levels of PRIDE skills. Further, Hannah’s use of PRIDE skills stabilized at the end of the intervention phase. When the maintenance phase began, there was a small but immediate decrease in Hannah’s use of PRIDE skills. However, throughout the maintenance phase, Hannah’s average use of PRIDE skills increased from the average of the intervention phase to an average of 29.2 (range: 20–33) PRIDE skills with some variability but no obvious trend. All data points in the maintenance phase overlapped with data points from the intervention phase. BC-Tau for the effect was 0.93 (SE = 0.07; 95% CI: [0.33, 0.99]), which is considered a very large effect.

Hannah used an average of 30.0 (range: 25–36) “Don’t skills” per session during the baseline phase, which trended downward for the first half of the baseline phase, then trended back upward. When the intervention phase began, there was no immediate or delated change in Hannah’s use of “Don’t skills, which averaged 33 (range: 25–46) “Don’t skills.” There was a slight downward but variable trend in Hannah’s use of “Don’t skills” across the intervention phase. When the maintenance phase began, there was a small but immediate increase in the number of “Don’t skills,” which trended downward throughout the maintenance phase with little variability. On average, Hannah used 29 (range: 18–38) “Don’t skills” during the maintenance phase. Several data points overlapped across all phases. The BC-Tau for this effect is −0.08 (SE = 0.30; 95% CI: [−0.57, 0.46]), which represents a small effect in the unintended direction (i.e., an increase in “Don’t skills”).

#### 3.1.2. Candance

Candance used an average of 8.4 (range: 3–16) PRIDE skills during baseline. There was moderate variability in her frequency of PRIDE skills; however, Candance’s use of PRIDE skills remained low at the end of the baseline phase. Candance’s use of PRIDE skills immediately increased following the “CDI Teach” session, although there was significant variation throughout the intervention phase but no obvious trend. On average, Candance used 22.8 (range: 3–40) PRIDE skills during the intervention phase. Three data points from the intervention phase overlapped with data points from the baseline phase. Candance’s increase in her use of PRIDE skills during the intervention phase was consistent with Hannah’s increase in the use of PRIDE skills, although Candance’s levels of PRIDE skills increased immediately while Hannah’s levels increased slowly. Candance’s use of PRIDE skills slightly decreased immediately at the beginning of the maintenance phase, but, on average, she used 21.2 (range: 14–25) PRIDE skills during the maintenance phase, which represents a slight decrease from the average of the intervention phase, and PRIDE skills were less variable in the maintenance phase than the intervention phase. Data patterns in the maintenance phase were consistent between Hannah and Candance. BC-Tau for this effect was 0.81 (SE = 0.13; 95% CI: [0.31, 0.95]), which represents a very large effect.

Candance’s use of “Don’t skills” trended upward across the baseline phase, and there was moderate variability in the number of “Don’t skills” used across sessions. On average, Candance used 36.0 (range: 23–50) “Don’t skills” in the baseline phase. Following the “CDI Teach” session, there was an immediate decrease in Candance’s use of “Don’t skills” followed by a large increase before her use of “Don’t skills” trended downward. On average, Candance used 33.9 (range: 26–55) “Don’t skills’ in the intervention phase. Data patterns between Candance and Hannah were similar in regard to level, trend, variability, and data overlap for the baseline and intervention phases. When the maintenance phase began, there was a large, immediate increase in the number of “Don’t skills,” which then immediately decreased to levels more similar to the baseline and intervention phase levels. There was an overall downward trend in Candance’s use of “Don’t skills” in the maintenance phase. On average, Candance used 46.3 (range: 21–92) “Don’t skills” per session in the maintenance phase. As with Hannah, there was a significant overlap of data points across all phases. BC-Tau for this effect was −0.01 (SE = 0.27; 95% CI: [−0.47, 0.45]), representing a small effect in the unintended direction.

#### 3.1.3. Melissa

During baseline, Melissa used an average of 13.1 (range: 9–18) PRIDE skills. Data had little variability and no obvious trend during baseline. Notably, the majority of Melissa’s PRIDE skills during baseline were reflections, as shown in Figure 3. Melissa’s use of other PRIDE skills remained low and stable throughout baseline. When the intervention phase began, there was a slight but immediate increase in Melissa’s use of PRIDE skills, which trended upward with limited variability throughout the intervention phase. During the intervention phase, Melissa used an average of 25.5 (range: 15–33) PRIDE skills per observation. Only one data point in the intervention phase overlapped with the baseline phase. Increases in the average level of PRIDE skills Melissa used during the intervention phase were consistent with increases from Hannah and Candance, and the increasing trend in Melissa’s PRIDE skills during intervention was consistent with Hannah’s increasing trend. When the maintenance phase began, there was a slight but immediate decrease in Melissa’s use of PRIDE skills. During the maintenance phase, Melissa’s used an average of 23 (range: 19–25) PRIDE skills, which was relatively stable throughout the maintenance phase and showed no obvious trend. All data points in the maintenance phase overlapped with data points from the intervention phase. Data patterns are consistent among all three caregivers in regard to a small but immediate decrease in PRIDE skills, minor variability throughout the maintenance phase, and overlap of all data points with the intervention phase, BC-Tau for this effect was 0.94 (SE = 0.06; 95% CI: [0.50, 0.99]), which indicates a very large effect.

Melissa’s use of “Don’t skills” was variable during the baseline phase with a general downward trend but averaging 18.9 (range: 9–35) “Don’t skills” per session. Following the implementation of the intervention, there was a very small and likely insignificant but immediate increase in Melissa’s use of “Don’t skills” which then trended upward with minor variability and averaged 24.2 (range: 15–38) “Don’t skills” per session. All but one of the data points in the intervention phase overlapped with data points in the baseline phase. When the maintenance phase began, Melissa’s use of “Don’t skills” decreased slightly but immediately, but overall trended upward with minor variability throughout the maintenance phase. On average, Melissa used 26.0 (range: 16–35) “Don’t skills” per session in the maintenance phase. As with the previous participants, data overlapped significantly across phases, but Melissa was the only participant with a decreasing trend in “Don’t skills” during baseline and an increasing trend during the intervention and maintenance phases. The BC-Tau for this effect was −1.00 (SE = 0.02; 95% CI: [1.00, 1.00]), indicating a very large effect in the unintended direction.

### 3.2. Child Engagement

**Figure 4:**
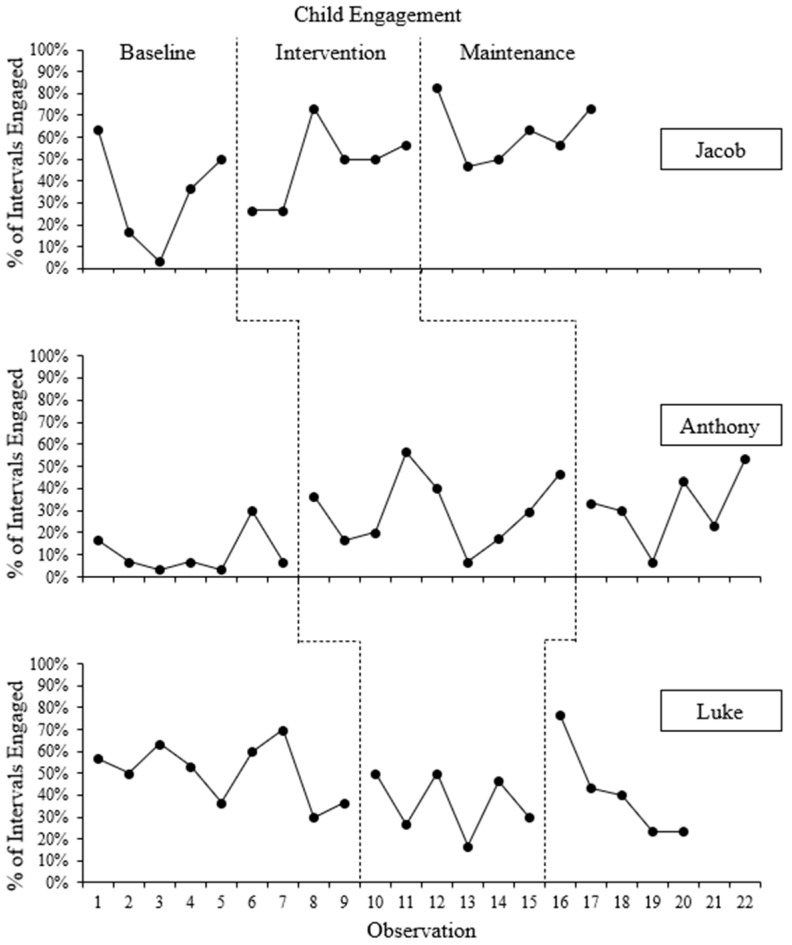
Percentage of intervals in which each child was engaged.

Child engagement data for all three participants is provided in Figure 4.

#### 3.2.1. Jacob

On average, Jacob was engaged with Hannah during the baseline phase 34.0% (range: 3.3–63.3%) of intervals in the baseline phase, although there was significant variability, but no obvious trend, in his levels of engagement. After the intervention phase began, there was an immediate decrease in child engagement, but Jacob increased his engagement to an average of 47% of intervals throughout the session. There was also significant variability (range: 26.7–73.3%) in Jacob’s engagement throughout the intervention phase, but data generally trended upward throughout the phase. All but one of the data points in the intervention phase overlapped with data points in the baseline phase. There was an immediate increase in the levels of Jacob’s engagement when the maintenance phase began, followed by an immediate decrease, then a generally upward trend. All but one of the data points in the maintenance phase overlapped with data points from the intervention phase. Further, while there was moderate variability in the maintenance phase, Jacob’s engagement increased to 62.1% (range: 46.7–82.8%), on average. The BC-Tau for this effect was 0.50 (SE = 0.29; 95% CI: [−0.11, 0.83]), representing a moderate effect.

#### 3.2.2. Anthony

Anthony’s engagement with Candance was variable throughout the baseline phase with no obvious trend but averaged 10.5% (range: 3.3–30.0%) of intervals. Following the “CDI Teach” session, there was an immediate increase in Anthony’s engagement, although his engagement continued to be variable (m = 30.0%; range: 6.7–56.7%) throughout the intervention phase. There was a notable increasing trend in Anthony’s engagement in the second half of the intervention phase, and about half of the data points in the intervention phase overlapped with data points from the baseline phase. Anthony’s data patterns are consistent with Jacob’s data patterns regarding increases in average levels of engagement across phases and variability within phases. Anthony’s engagement continued to be variable in the maintenance phase, with a small but immediate decrease in Anthony’s levels of engagement when the maintenance phase began, followed by a generally positive trend which average of 31.7% (range: 6.7–53.3%) of intervals engaged. All data points in the maintenance phase overlapped with the intervention phase. Data patterns regarding increases in the average levels of engagement across phases are consistent with Jacob’s engagement, although the degree of change in level was smaller. The BC-Tau for this effect was 0.75 (SE = 0.15; 95% CI: [0.24, 0.93]), representing a large effect of iPCIT on child engagement.

#### 3.2.3. Luke

As with the previous participants, Luke showed substantial variability in his engagement throughout all phases of the study. During the baseline phase, Luke was engaged during an average of 50.7% (range: 30.0–70.0%) of intervals, and his engagement trended downward with moderate variability throughout the baseline phase. When the baseline phase began, there was an immediate but small increase in Luke’s engagement, although throughout the intervention phase, Luke’s engagement averaged 36.7% (range: 16.7–50.0%) of intervals engaged, which is a decrease from average baseline levels. There was moderate variability but no obvious trend in Jacob’s levels of engagement during the intervention phase, and half of the data points in the intervention phase overlapped with data points in the baseline phase. Of note, during the four observations in which Luke’s mother attended an observation session, Luke engaged with his mother instead of Melissa during several intervals, but these intervals were coded as “not engaged” because Luke was not engaging with Melissa. However, there does not appear to be a significant difference in Luke’s engagement with Melissa among observation sessions when Luke’s mother did or did not attend, either in terms of level or variability. At the beginning of the maintenance phase, there was an immediate, large increase in the level of Luke’s engagement, but his engagement trended down sharply and steadily throughout the maintenance phase, with all but one data points overlapping with data from the intervention phase. On average, Luke was engaged during 41.3% (range: 23.3–76.7%) of intervals during the maintenance phase. Luke’s data patterns are inconsistent with those from Jacob and Anthony regarding direction of level change across phases and opposite trends in data. The BC-Tau for this effect was −0.48 (SE = 0.23; 95% CI: [−0.79, 0.04]), representing a moderate effect in the unintended direction.

### 3.3. Challenging Behavior

Caregiver-reported Problem and Intensity scores from the ECBI are reported for each participant in Table 1. Hannah and Melissa both reported a decrease in the number of challenging behaviors following intervention, with challenging behaviors no longer falling in the Clinically Significant range. In addition, Melissa also reported a decrease in the intensity of challenging behaviors following intervention. Candance did not complete the ECBI at the end of the study.

### 3.4. Social Validity

Results from the BIRS and C-CASS can be found in Table 2 and Table 3, respectively. On the BIRS, all raters indicated that PRIDE skills were a highly acceptable behavioral intervention for their children with adequate effectiveness and time to effectiveness. On the C-CASS, all raters indicated a high level of acceptability and satisfaction with the telehealth consultation process.

## 4. Discussion

The extant literature regarding iPCIT for children with ASD is limited to two case studies (Hong et al., 2018; Kawasaki & Kamo, 2020) and one small group design study (Matano et al., 2022). The present study was designed to test the implementation of iPCIT with families with autism as an intervention option to overcome treatment barriers associated with travel or cost and provide children with ASD preliminary skills that can facilitate more intensive therapy (i.e., joint attention).

The first research question asked whether there was a functional relation between iPCIT and an increase in caregiver PRIDE skills. All three caregivers demonstrated an increase in PRIDE skills following the intervention. This work is commensurate with previous findings that have shown iPCIT as an effective method for training caregivers to use PRIDE skills (Dufrene et al., 2024; Hong et al., 2018; Kawasaki & Kamo, 2020). Further, this study extends previous research by providing experimental evidence of this effect for caregivers of children with ASD within a single-case design framework, strengthening the generalizability and utility of the intervention.

It is noteworthy that Hannah’s levels of PRIDE skills demonstrated a slight increasing trend during baseline. Baseline trends can weaken causal inference as it is difficult to determine whether the increases following the implementation of the intervention were a result of the intervention itself or because of the passage of time. Although the overall baseline trend was increasing, there was a downward trend in Hannah’s use of PRIDE skills prior to the implementation of the intervention, which was followed by a change to an increasing trend and a higher rate of growth than was present in baseline. Further, the BC-Tau effect size, which provides a quantitative measure of treatment effect when there is a baseline trend, resulted in a very large effect. These additional considerations suggest that the implementation of the intervention still resulted in a treatment effect.

It is also noteworthy that Hannah did not demonstrate an immediate increase in her use of PRIDE skills, that Melissa’s initial increase in her use of PRIDE skills was very small, and that Candance’s large and immediate increase in her use of PRIDE skills was not sustained throughout the intervention phase. These findings suggest that the CDI Teach session, consisting of instructions, modeling with the interventionist, rehearsal and feedback, was not sufficient for consistent or sustained behavior change, and suggests that additional coaching, which was provided throughout the intervention phase, is necessary for caregivers to effectively use PRIDE skills at higher levels.

Caregivers may have increased their use of PRIDE skills as a result of reinforcement provided by the primary researcher during coaching and by the child in the form of improved behaviors. It is also possible that the provision of services via telehealth in the home may have contributed to caregivers’ success in increasing their use of PRIDE skills because caregivers were able to attend consistently without travel considerations, and skills were learned in the setting where they were expected to be used.

The second research question asked whether increases in PRIDE skills were maintained after intervention ended. Although all caregivers experienced a small decrease in PRIDE skills after the intervention was completed, levels of PRIDE skills remained higher than pre-intervention levels, with little or no overlap of data points. These results are consistent with previous research indicating PRIDE skills maintain over time, both for caregivers of children with ASD (Ginn et al., 2017; Masse et al., 2016) and when delivered to caregivers via iPCIT (Dufrene et al., 2024). This finding extends the literature by demonstrating that caregivers of children with ASD also maintain their use of PRIDE skills when training was provided via iPCIT. Further, these results extend the literature by replicating the maintenance effects of previous studies, increasing confidence in caregivers’ ability to maintain their use of these skills after training. It is possible that teaching skills to parents in the home promotes maintenance of skills because environmental stimuli (e.g., the room where the skills were learned, the handout given to caregivers) served as prompts for caregivers to continue to use the skills. It is also possible that caregivers’ use of PRIDE skills continued to be reinforced after the intervention ended by improvements in child behaviors, such as increased engagement or decreased challenging behaviors.

The third research question asked whether a functional relation existed between iPCIT and a decrease in caregiver’s use of “Don’t skills” if they were not taught to avoid them. Effect size data indicated that only one caregiver (Hannah) used fewer “Don’t skills,” on average, during the intervention and maintenance phases. However, visual analysis indicated a great deal of overlap between baseline and intervention and maintenance phases, suggesting that there may not be a practical difference in “Don’t skills.” Further, the other two caregivers (Candance and Melissa) used more “Don’t skills” in later phases than they did during baseline, and Melissa’s use of “Don’t skills” trended upward during the intervention and maintenance phases.

It is possible that caregivers were focused on increasing their overall attempts to engage with their children, which may have resulted in an overall increase in caregiver statements to their child. Specifically, caregivers were aware that one purpose of the study was to measure changes in their child’s engagement, so it is possible that caregivers engaged in behaviors that they felt were likely to increase their child’s engagement (e.g., asking more questions). Anecdotally, caregivers often asked questions that allowed them to use a PRIDE skill (e.g., reflection—Caregiver: “What color is this?” Child: “Purple.” Caregiver: “It is purple!”) or gave instructions to attend to the toys with which the caregiver was playing (e.g., “Look at this!”). Caregivers may not have demonstrated improvements in “Don’t skills” simply because they were not trained to do so. It may be possible that, with training, caregivers’ use of “Don’t Skills” would have decreased. Thus, direct training on the avoidance of “Don’t Skills,” in addition to training to use PRIDE skills, should be viewed as an essential component of PCIT (Eyberg & Funderburk, 2022).

The fourth research question asked whether there was a functional relation between iPCIT and an increase in child engagement, which plays a critical role in the development of literacy, language, and communication (Adamson & Dimitrova, 2014), particularly for children with ASD (Bottema-Beutel, 2016). Although Jacob and Anthony exhibited higher levels of engagement in the intervention phase than in the baseline phase, there was significant variability in levels of engagement across observations. Further, Luke showed the opposite pattern, with less engagement during the intervention phase compared to the baseline phase.

There are several possible reasons why Luke’s engagement with his caregiver may have decreased across phases. Perhaps the most likely reason is that Melissa prohibited the use of electronics during the intervention and maintenance phases. Specifically, during the baseline phase, Melissa often allowed Luke to trace pictures by placing a piece of paper over the screen of a tablet; however, when Luke requested to use the tablet for this activity during the intervention and maintenance phases, Melissa denied his request, which often led to challenging behaviors (e.g., hitting or ignoring) instead of engagement. A second possible explanation is that Melissa’s use of “Don’t skills” increased across the intervention phase, which may have been aversive to Luke, resulting in less engagement. A third possible explanation is that Melissa’s fall and subsequent mobility limitations may have altered the nature of their relationship, which may have led to a decrease in Luke’s engagement. For example, Melissa’s limited mobility may have led her to engage with Luke less frequently, which in turn may have decreased the frequency with which he engaged with Melissa. Alternatively, the novelty of seeing Melissa wearing casts on both arms may have influenced Luke to behave differently around Melissa. A fourth explanation is that Melissa was unable to use many imitations due to her limited mobility, and it is possible that imitations are a critical component in improving child engagement. Fifth, Luke and Melissa had the lowest rate of practice between sessions and the fewest number of days between sessions, which may have provided fewer overall opportunities for Luke to adjust to Melissa’s increasing use of PRIDE skills. Finally, it is possible that variables outside of the study account for the differences.

Overall, iPCIT was associated with an increase in child engagement for two out of three participants. This finding advances the literature by extending the possibility that child engagement may also be improved when PCIT is delivered via the internet. However, further research should be conducted to clarify the nature of the functional relation between PCIT/iPCIT and child engagement. If iPCIT is a valid, accessible intervention that has a positive effect on the engagement of children with ASD, it could be used to provide services to families that otherwise would not be able to access treatment (Barnett et al., 2021; Fleming et al., 2017).

The fifth research question asked whether increases in child engagement maintained after iPCIT sessions ended. For both children whose engagement increased during intervention, levels of engagement during maintenance either increased from (Jacob) or remained at similar levels (Anthony) as the intervention phase. Although Luke’s average level of engagement was higher during the maintenance phase, levels of engagement trended downward across the phase. Engagement during the maintenance phase may represent a different response than engagement during the intervention phase because caregivers have demonstrated mastery levels of PRIDE skills during the maintenance phase. Thus, engagement during the maintenance phase represents a child’s response to a higher dose of PRIDE skills than during the maintenance phase, when caregivers were still learning to implement PRIDE skills. This was a novel finding because maintenance of child engagement following the end of iPCIT has not previously been examined in research literature. Although increased levels of child engagement were found for both participants who initially showed improvements, given that only two participants initially demonstrated an increase in levels of engagement during iPCIT, replications should be conducted to confirm these findings.

The sixth research question asked whether iPCIT would result in a decrease in caregiver-reported challenging behaviors in children with ASD, as measured by the ECBI. Only two caregivers completed the ECBI at both the beginning and end of the study, but both reported a decrease in challenging behaviors from the clinical range to the subclinical range. Thus, it appears that CDI-only iPCIT has the potential to improve caregiver-reported challenging behaviors in children with ASD. Previous research (Hong et al., 2018; Kawasaki & Kamo, 2020; Matano et al., 2022) has suggested that iPCIT may effectively reduce challenging behaviors in this population, but none of these studies have examined this effect in the context of the CDI phase only. In addition, the mastery criteria for caregivers were reduced in the current study, and improvements in child challenging behaviors were reported for both children. This suggests that the intense mastery criteria that have been associated with PCIT attrition (Ufford et al., 2022) may not be necessary to achieve improvements in challenging behaviors.

The seventh and eighth research questions asked whether caregivers perceived PRIDE skills as a socially valid strategy to improve their child’s engagement and whether they perceived iPCIT as a socially valid parent training modality. All three caregivers indicated that PRIDE skills were highly acceptable as an intervention strategy for their child’s engagement and high levels of acceptability and satisfaction with the iPCIT process. Further, Hannah reported anecdotally that after completing the study, she would continue to use PRIDE skills in her interactions with Jacob because she felt it had resulted in increases in Jacob’s attempts to communicate and play with Hannah. These results are commensurate with previous findings which have shown that caregivers typically report high levels of satisfaction with PCIT, whether provided in person (Allen et al., 2022; Zlomke & Jeter, 2020) or via internet (Fleming et al., 2021). However, previous measures of social validity related to PCIT have measured overall satisfaction with both the treatment outcomes and therapeutic process together. This study extends the literature because it separately assessed the social validity of the intervention and the therapeutic process and confirmed that caregivers generally reported satisfaction with both. Further, previous studies assessing the social validity of iPCIT/PCIT have assessed the social validity of the entire PCIT protocol; the current study assessed the social validity of the CDI phase alone and used a lower mastery criterion than previous studies and still found high caregiver satisfaction with the intervention and therapeutic process.

### 4.1. Implications for Practice

A few implications for practice are noteworthy. First, these results add to a growing body of research that suggests that iPCIT is an effective and acceptable modality for teaching caregivers of children with ASD to use PRIDE skills. Delivering parent training interventions over the internet can allow clinicians to provide needed services to families without regard to barriers such as transportation distance or costs, as long as families have reliable access to the internet. Qualified providers of family behavioral health services should consider the use of iPCIT when doing so would benefit families who might otherwise have limited access to family behavioral health services. Second, because caregivers did not decrease their use of “Don’t skills” as they increased their use of PRIDE skills, practitioners should ensure that iPCIT training includes instruction on increasing use of PRIDE skills and decreasing use of “Don’t skills” to avoid potentially aversive interactions between caregivers and children.

Third, high mastery criteria, and resultingly long treatment phases have been cited as a potential reason for high attrition rates among PCIT families (Ufford et al., 2022). This study decreased the CDI mastery criteria for caregivers from ten each of labeled praise statements, reflections, and behavioral descriptions (Eyberg & Funderburk, 2022) to three each of labeled praise statements, reflections, imitations, and behavioral descriptions. As a result, caregivers were able to achieve mastery criterion in as little as two or three coaching sessions, and effects were still found on child challenging behavior, with mixed effects on child engagement. This suggests that meeting such high mastery criteria may not be necessary for treatment outcomes in PCIT. Delivering iPCIT with an abbreviated protocol, as in the present study, could also be beneficial to families who are on intervention waitlists; because early intervention is critical in neurodevelopmental disorders such as ASD, empowering caregivers to help their children build skills while awaiting access to additional services takes advantage of early critical periods of development (Scudder et al., 2019). Fourth, increases in child engagement may be a valid outcome of iPCIT when implemented with children with ASD and their caregivers. The current study demonstrated that CDI-only iPCIT resulted in increased levels of child engagement for two out of three participants. Although confounding factors may explain the lack of improvement for the third participant, it is noteworthy that other studies measuring the effects of PCIT on child engagement (Furukawa et al., 2018; Han, 2021; Rigg, 2016) had limitations in demonstrating this effect. Thus, practitioners should proceed with caution in implementing PCIT/iPCIT to increase child engagement. Further research should also be conducted to clarify the relationship between PCIT/iPCIT and child engagement for children with ASD.

Finally, providing internet-delivered parent training services presents unique challenges that are often not present when providing services in person. Handouts must be provided to caregivers in advance of sessions, and if they are delivered electronically, caregivers may need the capability to print handouts prior to session if they are to be used during the session. Caregivers should also receive instruction on how to prepare an appropriate space for therapy sessions, including the use of PCIT-appropriate toys, a large enough space for play to occur naturally, and appropriate camera placement to allow the provider adequate vision of the play space. In the present study, time was allotted prior to the initial baseline session to assist the caregiver in this preparation, so providers may desire to schedule additional times for sessions early in the process to accommodate this preparation. Further, there were times when the videoconferencing software failed for various reasons (e.g., poor internet connection, malfunctioning hardware), so providers should have backup hardware available for their own use and a plan to communicate with caregivers in the event the caregiver loses access to the video conference. Additional and more comprehensive suggestions for providing PCIT in a telehealth format are provided by Peskin et al. (n.d.).

### 4.2. Limitations and Future Directions

A number of limitations of the current study should be addressed. First, as with all single-case designs, the generalizability of this study may be limited to populations similar to the included participants. Still, the participants represent a variety of demographic groups, including children in early and middle childhood, two-parent and multigenerational households, White and Black families, children with varied language levels, and families with a single child and multiple children. However, all families had pre-existing access to adequate technology for telehealth services, all families were college-educated, all participating caregivers were female, all participating children were male, and various cultural backgrounds, which are already underrepresented in PCIT literature, were not included. Thus, results should not be generalized beyond the included population. Future studies should seek participants from other populations, particularly those who are already underrepresented in PCIT research.

Second, all participants in this study received additional services, which were not controlled in the study and which were not always consistent from one participant to another or from one phase to the next. Specifically, Jacob received speech therapy services throughout the study, Anthony received speech therapy services throughout the study and initiated occupational therapy services and pharmacotherapy (i.e., guanfacine for ADHD) between the intervention and maintenance phases, and Luke received speech therapy services at school, but not during the course of the study, which was conducted during the summer break. Any effects that result from these services outside the study could have confounded the results of this study. Future research may seek to control or standardize other therapeutic services that participants are receiving to minimize the effects of confounding variables.

Third, the effects of Melissa’s medical challenges required several adaptations to the study protocol for Melissa and Luke. First, as reported above, Melissa was unable to imitate many of Luke’s behaviors. Second, also described above, Luke’s mother sat in on some play sessions when she was available to do so, which resulted in Luke engaging with his mother instead of his grandmother. Third, Melissa and Luke had limited opportunities to practice PRIDE skills due to Melissa spending time in the hospital or resting in bed. Fourth, it is possible that Melissa’s medical condition may have changed Melissa and Luke’s relationship in intangible ways that may have affected the data, beginning at the start of the intervention phase.

Fourth, because Candance did not complete the ECBI at post-intervention, it is not possible to determine whether or how Anthony’s challenging behaviors may have changed. Notably, both Anthony’s Problem and Intensity scores fell just below the clinical cut-off, suggesting that he did not engage in significant levels of challenging behaviors, but it is not possible to determine whether iPCIT may have still resulted in decreased levels of challenging behaviors. Furthermore, the ECBI was the only measure of challenging behavior collected in this study, which may be subject to bias as it was completed by the caregivers who participated in the intervention targeting challenging behavior. Future studies may wish to include direct observations of challenging behaviors in addition to, or instead of, caregiver-report measures.

Fifth, some researchers (e.g., Carr, 2005; Kennedy, 2022) argue that nonconcurrent multiple baseline designs, as were used in the present study, are subject to additional threats to internal validity, such as confounds due to maturation, history, or observer drift, compared to concurrent designs. Alternatively, others (Harvey et al., 2004; Morin et al., 2024; Slocum et al., 2022) argue that nonconcurrent designs are uniquely robust designs for internal and external validity due to their ability to measure intervention effects across a wider range of time while engaging in ethical, practice-based research, as treatment is not withheld from families until a sufficient number of participants can be recruited. Future researchers may wish to replicate this study using a concurrent design to address weaknesses of nonconcurrent designs.

## 5. Conclusions

The aim of this study was to determine whether iPCIT is an effective treatment for families with children with ASD. Results indicated that iPCIT resulted in an increase in PRIDE skills for all caregivers, increased child engagement for two out of three children, and decreased challenging behaviors for two of three children. Additionally, all caregivers rated PRIDE skills as an acceptable and socially valid intervention for their children and rated iPCIT as an acceptable and socially valid treatment modality. These results extend previous literature on iPCIT for children with ASD by providing experimental evidence of iPCIT in this population within a single-case design framework, direct measures of both parent skills and child engagement, and showing that an increase in caregiver PRIDE skills does not result in a concomitant decrease in “Don’t skills” when they are not explicitly taught. Overall, these findings suggest that iPCIT may be an effective intervention for improving challenging behaviors and child engagement in children with ASD.

## Figures and Tables

**Table 1:** Pre- and Post-Intervention ECBI Scores.

Child	Intensity		Problem	
	Baseline	Maintenance	Baseline	Maintenance
Jacob	127 (59)	128 (59)	18 (64) *	8 (51)
Anthony	129 (59)	-	14 (59)	-
Luke	130 (59)	89 (48)	24 (72) *	5 (47)

Note. Scores outside parentheses are raw scores and scores inside parentheses are T-scores. * = Clinically Significant levels of problem behavior.

**Table 2:** Behavior Intervention Rating Scale.

Caregiver	Acceptability	Effectiveness	Time to Effectiveness
Hannah	5.27	4.57	6.00
Candance	5.47	4.71	4.00
Melissa	5.40	5.14	5.00
Average	5.38	4.81	5.00

Note. Scores range from 1 (strongly disagree) to 6 (strongly agree).

**Table 3:** Clinical Consultation Acceptability and Satisfaction Scale.

Caregiver	Average C-CASS Score
Hannah	4.83
Candance	5.00
Melissa	5.00
Average	4.94

Note. Scores range from 0 (strongly disagree) to 5 (strongly agree).

## Data Availability

The raw data supporting the conclusions of this article will be made available by the authors on request.

## Abbreviations

The following abbreviations are used in this manuscript:

95% CI	95% Confidence Interval
ADHD	Attention-deficit Hyperactivity Disorder
ASD	Autism Spectrum Disorder
BC-Tau	Baseline Corrected Tau
BIRS	Behavior Intervention Rating Scale
C-CASS	Clinical Consultation Acceptability and Satisfaction Scale
CDI	Child-directed Interaction
DPICS	Dyadic Parent–Child Interaction Coding System
ECBI	Eyberg Child Behavior Inventory
FERPA	Family Educational Rights and Privacy Act
HIPAA	Health Insurance Portability and Accountability Act
IOA	Interobserver agreement
iPCIT	Internet-delivered Parent Child Interaction Therapy
PCIT	Parent–child Interaction Therapy
PDI	Parent-directed Interaction
PRIDE	Praise, Reflections, Imitations, Descriptions, Enjoyment/Enthusiasm
SE	Standard Error
